# KEAP1‐NRF2/HO‐1 Pathway Promotes Ferroptosis and Neuronal Injury in Schizophrenia

**DOI:** 10.1002/brb3.70311

**Published:** 2025-02-28

**Authors:** Feng Zhu, Tangqun Dan, Shuguang Hua

**Affiliations:** ^1^ Department of Psychiatry The Second Affiliated Hospital of Hubei University of Science and Technology Xianning Hubei China

**Keywords:** ferroptosis, hiPSC‐derived cortical interneurons, KEAP1‐NRF2/HO‐1, neuronal injury, oxidative stress, schizophrenia

## Abstract

**Background:**

This study investigates the role of the KEAP1‐NRF2/HO‐1 signaling pathway in inducing ferroptosis and contributing to neuronal damage in schizophrenia.

**Methods:**

We retrieved schizophrenia‐related data and ferroptosis‐related genes from the RNA microarray dataset GSE27383 and FerrDB database, respectively. Bioinformatics data identified KEAP1 as a downregulated gene, which was validated using qRT‐PCR and Western blot. We assessed intracellular Fe^2^⁺ content, MDA levels, GSH, and GPX4 in the prefrontal cortex and peripheral blood mononuclear cells (PBMCs) of patients with schizophrenia. Cortical interneurons (cINs) were generated from human‐induced pluripotent stem cells (hiPSCs) of patients with schizophrenia and used to explore KEAP1 alterations during neurodevelopment. In addition, KEAP1 overexpression was induced in cINs via transfection with pcDNA KEAP1. The intracellular Fe⁺ levels, oxidative stress indicators, lipid peroxidation, and inflammatory cytokines were measured after transfection. To investigate molecular mechanisms, KI696—a high‐affinity probe that disrupts the KEAP1–NRF2 interaction—was applied, and changes in oxidative stress, lipid peroxidation (C11‐BODIPY staining), iron metabolism, and inflammatory pathways were evaluated.

**Results:**

Patients with schizophrenia exhibited underexpression of KEAP1, a key regulator of ferroptosis, along with elevated intracellular Fe^2^⁺ levels and increased MDA concentrations, indicating enhanced lipid peroxidation and oxidative stress. Reduced GPX4 activity and GSH levels were also observed, suggesting an increased susceptibility to ferroptosis. To further explore this, cINs derived from hiPSCs of patients with schizophrenia were studied. These cells showed decreased KEAP1 expression. Overexpression of KEAP1 in cINs led to a reduction in intracellular Fe^2^⁺ concentrations and oxidative damage, highlighting KEAP1's regulatory role in ferroptosis. In addition, treatment with KI696 induced significant alterations in pathways related to oxidative stress, iron metabolism, antioxidant defenses, and inflammation.

**Conclusion:**

Our findings indicate that the KEAP1‐NRF2/HO‐1 pathway contributes to ferroptosis and neuronal injury in schizophrenia.

## Graphical abstract

KEAP1‐NRF2/HO‐1 pathway regulates ferroptosis, oxidative stress, iron metabolism, and inflammation contributing to neuronal damage in schizophrenia.

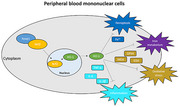

## 1 Introduction

Schizophrenia (SCZ) is a persistent mental condition defined by delusions, hallucinations, disorganized behavior, and cognitive difficulties (McCutcheon et al. 2023; Winship et al. 2019). The causes of SCZ are complex, encompassing genetic, neurological, and environmental variables, however, the specific mechanism is yet unclear (Schmitt et al. 2023; Tandon et al. 2024). Recent studies have increasingly recognized oxidative stress and neuroinflammation as central contributors to the pathophysiology of SCZ (Chaves et al. 2024; Chen et al. 2023; Mosquera et al. 2024; Sawa and Sedlak 2016).

One critical pathway implicated in the regulation of oxidative stress is the KEAP1‐NRF2/HO‐1 pathway, which functions as a protective mechanism against oxidative damage (Yi et al. 2024; Zhang et al. 2018). Nuclear factor erythroid 2‐related factor 2 (NRF2) is a crucial transcription factor that regulates the expression of antioxidant proteins and protects cells from inflammation‐induced oxidative damage (Guo et al. 2024; X. Wang, Liu, et al. 2021). Under normal conditions, NRF2 is bound in the cytoplasm by KEAP1. However, during oxidative stress, NRF2 dissociates from KEAP1, moves into the nucleus, and activates genes responsible for antioxidant defense, including heme oxygenase‐1 (HO‐1) (Bellezza et al. 2018; Luo et al. 2022; Suzuki et al. 2023). HO‐1 is an enzyme activated by stress, which protects cells from oxidative damage by degrading heme, a pro‐oxidant molecule, into biliverdin, free iron, and carbon monoxide (An et al. 2021; Guo et al. 2023; L. Wang, Lou, et al. 2023).

Apart from its role as an antioxidant, the KEAP1‐NRF2‐HO‐1 pathway is involved in ferroptosis, a regulated cell death driven by iron‐dependent lipid peroxidation (X. Wang, Li, et al. 2024; Wei et al. 2021). Ferroptosis has been identified as a contributing factor in several neurodegenerative and psychiatric disorders, including SCZ, due to its strong association with iron dysregulation and oxidative stress (Liu et al. 2024; Ryan et al. 2023; Y. Wang, Li, et al. 2024; Y. Wang, Wu, et al. 2023). Iron accumulation and oxidative damage are key contributors to neuronal injury, which is believed to underlie some of the cognitive impairments observed in SCZ (Mohan et al. 2024; C. Wang, Chen, et al. 2022; Xu et al. 2024).

Given the connection between oxidative stress, ferroptosis, and SCZ, investigating the KEAP1‐NRF2/HO‐1 pathway in this context is critical for understanding the molecular mechanisms driving neuronal vulnerability in SCZ. We explored the involvement of KEAP1‐NRF2/HO‐1 pathway in initiating ferroptosis and its potential impact on neuronal injury in SCZ. Our aim is to enhance the understanding of the mechanism underlying neuronal injury and SCZ pathophysiology.

## 2 Materials and Methods

### 2.1 Bioinformatics Analysis

The SCZ RNA microarray dataset GSE27383 was utilized for this analysis, resulting in the identification of 922 differentially expressed genes (DEGs). A list of 264 ferroptosis‐related genes was gathered from the FerrDb database (http://www.zhounan.org/ferrdb/current/). These datasets were then cross‐referenced to filter out DEGs associated with ferroptosis. Differential expression analysis was conducted on the GSE27383 dataset using the GEO2R tool, which allowed for the comparison of gene expression profiles.

### 2.2 Patients Samples

Tissue samples were collected from 15 SCZ patients and 15 healthy controls. Inclusion criteria for the SCZ cohort required participants to have a confirmed diagnosis of SCZ, between the ages of 18 and 50 years, and have the capacity to provide informed consent. SCZ patients were excluded based on the following criteria: (1) diagnosis of other mental disorders or use of antipsychotic drugs within the past month; (2) prior treatment with physiotherapy or psychotherapy; (3) presence of systemic diseases, including immune, endocrine, or metabolic disorders; (4) history of alcohol or substance abuse or dependence; (5) risk of self‐harm or harm to others; and (6) pregnancy or lactation. Prefrontal cortex tissue was collected via biopsy under local anesthesia and immediately preserved in RNA stabilization solution (Qiagen, Germany) to prevent RNA degradation. Peripheral blood mononuclear cells (PBMCs) were separated from blood using a Ficoll‐Paque PLUS density gradient. Following a 30‐min centrifugation at 400×, the PBMC layer was carefully removed from the blood samples and washed thoroughly with phosphate‐buffered saline. Samples were then stored in liquid nitrogen after 24 h for long‐term preservation. We submitted and received approval of this protocol (HU2022_GHXZ) from Hubei University of Science and Technology.

### 2.3 Quantitative Real‐Time Reverse‐Transcription Polymerase Chain Reaction

Total RNA was extracted and reverse‐transcribed into cDNA. The reaction mixture, which included cDNA, SYBR Green PCR Master Mix, gene‐specific primers, and nuclease‐free water, was prepared and loaded into PCR plate wells. Gene expression levels were quantified using GAPDH as the internal control for normalization. Sequences of primers of KEAP1 and GPX4 are shown in Table 1.

**TABLE 1:** Primer sequences of KEAP1, GPX4, and GAPDH.

KEAP1 Fwd:	AAGAGGATGAGGAGGAGGAAAG
Rev:	CTTCTGGGGATCTGAGGCTG
GPX4: Fwd:	GACATCAGGAGAACGGAAGC
Rev:	TCGAGTCCGAAAGGAGACAA
GAPDH: Fwd:	GAAGGTGAAGGTCGGAGTCA
Rev:	GACAAGCTTCCCGTTCTCAG

### 2.4 Western Blot

Proteins acquired after lysis of cells were carefully separated on SDS‐PAGE and then shifted onto nitrocellulose membranes that were incubated with primary antibodies specific to β‐tubulin (Cell signaling#2146, 1:900), SOX6 (Proteintech, Cat. #14010‐1‐AP, 1:25), NRF2 (Proteintech#80593‐1, 1:50), HO‐1 (Novus Bio# NBP1‐97507, 1:75), GAPDH (Santa Cruz#sc32233, 1:20), GPX4 (Thermo Fischer#MA5‐32827, 1:30), and KEAP1 (Cell signaling#8047). After washing, HRP‐conjugated secondary antibodies (Biotechne antirabbit# HAF008 , 1:300 and anti‐mouse# HAF007 , 1:500) were applied on these membranes for 1 h. The protein bands were visible with chemiluminescence detection technique (Pierce Biotechnology).

### 2.5 Iron Reduction Assay

Prefrontal cortex tissues and PBMCs were isolated from both SCZ patients and healthy control subjects. The tissue and cell samples were homogenized in chilled phosphate‐buffered saline and subjected to centrifugation to eliminate insoluble material. The resulting supernatant was used for the assay. To facilitate the reduction of Fe^3^⁺ to Fe^2^⁺, an iron‐reducing reagent was added to the samples and incubated for 30‐min at 37°C. Afterward, an iron‐specific probe was introduced into samples which remained incubated for another 60 min in the dark to avoid light‐induced degradation. To determine the iron concentration, the absorbance was measured at 593 nm using a microplate reader.

### 2.6 Quantification of Malondialdehyde and Glutathione Levels

Levels of malondialdehyde (MDA) (Sigma#MAK568) and glutathione (GSH) (Sigma#MAK440) were measured with these kits. For the MDA assay, the samples were mixed with an MDA‐specific probe and incubated at 95°C for 2 h and absorbance was recorded at 532 nm to quantify MDA levels. In the GSH assay, the samples were first deproteinized and then incubated with the GSH detection probe for 30 min to 1 h. GSH concentrations were determined by measuring fluorescence at 490 and 520 nm, respectively.

### 2.7 Enzyme‐Linked Immunosorbent Assay

Enzyme‐linked immunosorbent assay (ELISA) kits (NEB#10754C) were used to evaluate GPX4 activity and inflammatory cytokine levels. In brief, the samples were loaded into microplate wells that had been pre‐coated with antibodies specific to the target proteins. Subsequently, a secondary antibody was added to the wells. After series of incubation and washing steps, a substrate solution was introduced, allowing color development proportional to the concentration of the target proteins. The reaction was stopped, and the absorbance at 450 nm was determined using a microplate reader.

### 2.8 Generation of Human‐Induced Pluripotent Stem Cells (hiPSCs) From Patients

Skin fibroblasts were collected from 15 SCZ patients who were part of a cooperative care model, designed to ensure holistic support throughout the process. The cooperative nursing approach included interdisciplinary teams of physicians and nurses who developed individualized care plans, monitored patient progress, and provided guidance to patients and their families on the research procedures. Emotional support and educational initiatives were incorporated to promote positive patient interactions and reduce anxiety during the study. Following informed consent, skin biopsies were obtained from the patients, and fibroblasts were isolated. The fibroblasts were reprogrammed into induced iPSCs with the help of reprogramming factors Oct4, Sox2, Klf4, and c‐Myc using standard protocols. The reprogrammed cells were cultured and expanded under feeder‐free conditions to maintain pluripotency. These iPSCs were subsequently used to explore alterations during neurodevelopment.

### 2.9 Differentiation of iPSCs Into Cortical Interneurons

The differentiation of iPSCs into neural progenitor cells (NPCs) was achieved using a dual SMAD inhibition method. This was achieved by treating the cells with Noggin (Sino Biological, Cat. #10267‐HNAH) and SB431542 (Tocris, Cat. #1614) to block the TGF‐β and BMP signaling pathways. The NPCs were then directed toward medial ganglionic eminence (MGE)‐like progenitors through modulation of the sonic hedgehog (SHH) signaling using recombinant SHH protein (R&D Systems, Cat. #1845‐SH‐025) and activation of the WNT pathway with CHIR99021 (Stem cells, Cat. #99021). Further differentiation into cortical interneurons (cINs) was facilitated in the presence of neurotrophic factors. The cultured cINs underwent maturation over several weeks, aided by the administration of brain‐derived neurotrophic factor (Life sciences# BLC‐06003), glial cell line‐derived neurotrophic factor (Sigma# SRP3200), and ascorbic acid to enhance neuronal survival and maturation. The identity of the cINs was validated through immunofluorescence analysis.

### 2.10 Immunofluorescence

cINs were seeded in 48‐well plates (1 × 10⁶ cells) and grown to approximately 70% confluence. Afterward, they were fixed with 4% paraformaldehyde, washed thrice, and incubated with primary antibodies. This includes β‐tubulin (Proteintech, Cat. #10094‐1‐AP) and SOX6 (Proteintech, Cat. #14010‐1‐AP). The cells were treated for 1 h with secondary antibodies that were fluorophore‐conjugated (Alexa Fluor488 for tubulin, Invitrogen, Cat#A12379 and Alexa Fluor568 for SOX6, Fisher scientific, cat#A12380). To visualize the cell nuclei, DAPI was used for nuclear staining. Fluorescence microscopy (Nikon) was used to examine the expression of neuronal marker β‐tubulin and cortical cIN marker SOX6.

### 2.11 Transfection of Cortical Interneurons

Cells were transfected using Lipofectamine 2000 (Invitrogen, USA). Two plasmids were used for transfection: pcDNA3.1 (empty vector control) and pcDNA KEAP1 (overexpression vector for KEAP1) and divided into normal, SCZ, SCZ‐pCDNA3.1, and pcDNA KEAP1. Following transfection, the cells were cultured for 48 h to allow gene expression, and transfection efficiency was assessed by RT‐qPCR and Western blot. KI696 was added to the culture media of pcDNA KEAP1 at a concentration of 1 µM based on previous study to disrupt the KEAP1–NRF2 interaction (Weiss‐Sadan et al. 2023) for 48 h, after which the cells were harvested for further experiments.

### 2.12 Assessment of Lipid Peroxidation

cINs once confluent (1 × 10^6^) in 48‐well plate were washed with phosphate buffer saline to eliminate leftover media from the cells. To measure lipid peroxidation, they were treated with 2 µM C11‐BODIPY(581/591) at 37°C. After incubation, the cINs were rinsed with washing buffer to remove any unbound dye. The cells were trypsinized, centrifuged, and resuspended in PBS; lipid peroxidation was immediately analyzed with excitation at 488 nm.

### 2.13 Statistical Analysis

Data are reported as mean ± SD. Group comparisons were conducted using Student's *t*‐test for two groups or ANOVA for multiple groups. When ANOVA revealed significant differences, Tukey's post hoc test was employed to compare individual groups. All experiments were performed in triplicate, and the results were analyzed with SPSS software.

## 3 Results

### 3.1 KEAP1 is Downregulated in SCZ

**FIGURE 1:**
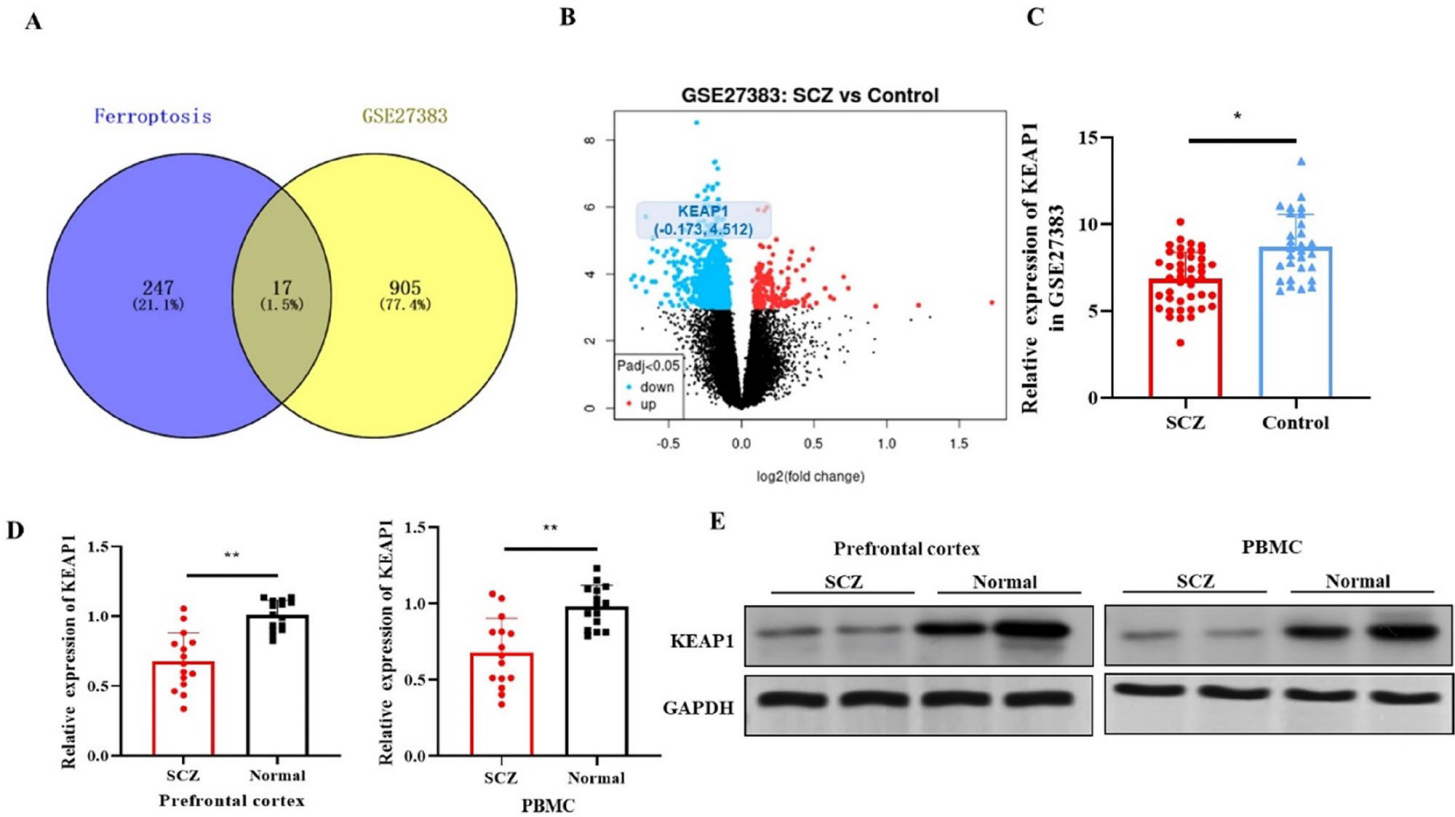
KEAP1 is underexpressed in SCZ. (A) Venn diagram showed overlap between 922 DEGs from the SCZ RNA microarray dataset GSE27383 and 264 ferroptosis driver genes from the FerrDb database. (B) Volcano plot analysis from the GSE27383 dataset using the GEO2R online tool. DEGs were selected based on *p* < 0.05 and |log FC| > 0.1, with downregulated genes (log FC < 0) shown in blue and upregulated genes (log FC > 0) in red. (C) KEAP1 was identified as a downregulated ferroptosis driver gene in the GSE27383 dataset. (D–E) mRNA and protein levels of KEAP1 in prefrontal cortex samples and PBMCs from 15 SCZ patients and 15 healthy controls. *p < 0.05 and **p < 0.01 show significance when compared to control group.

To examine the expression of ferroptosis‐related genes in SCZ, we conducted a comparative analysis using the SCZ RNA microarray dataset GSE27383 and a dataset of 264 known ferroptosis driver genes from the FerrDb database. The intersection of these two datasets identified 17 overlapping genes (Figure 1A), indicating a potential role of ferroptosis in the pathology of SCZ. Among these genes, KEAP1, a key regulator of the NRF2 pathway, was found to be significantly downregulated (log FC = −0.173, *p* < 0.05), as shown in the volcano plot (Figure 1B). The GSE27383 dataset analysis showed that SCZ patients have considerably lower levels of KEAP1 expression as compared to controls (Figure 1C). Further analysis via quantitative real‐time reverse‐transcription polymerase chain reaction (qRT‐PCR) and Western blot using samples from 15 SCZ patients and 15 healthy controls confirmed the reduced expression of KEAP1 in the prefrontal cortex and PBMCs of SCZ patients (Figures 1D,E). Quantification of KEAP1 expression through qRT‐PCR showed lower levels in the SCZ group (Figure 1D). In addition, Western blot analysis corroborated these findings, demonstrating reduced protein levels of KEAP1 in both the prefrontal cortex and PBMC samples from SCZ patients (Figure 1E). These results suggest that the ferroptosis driver KEAP1 is consistently underexpressed in individuals with SCZ.

### 3.2 Ferroptosis Is Active in the Prefrontal Cortex and PBMCs of SCZ Patients

**FIGURE 2:**
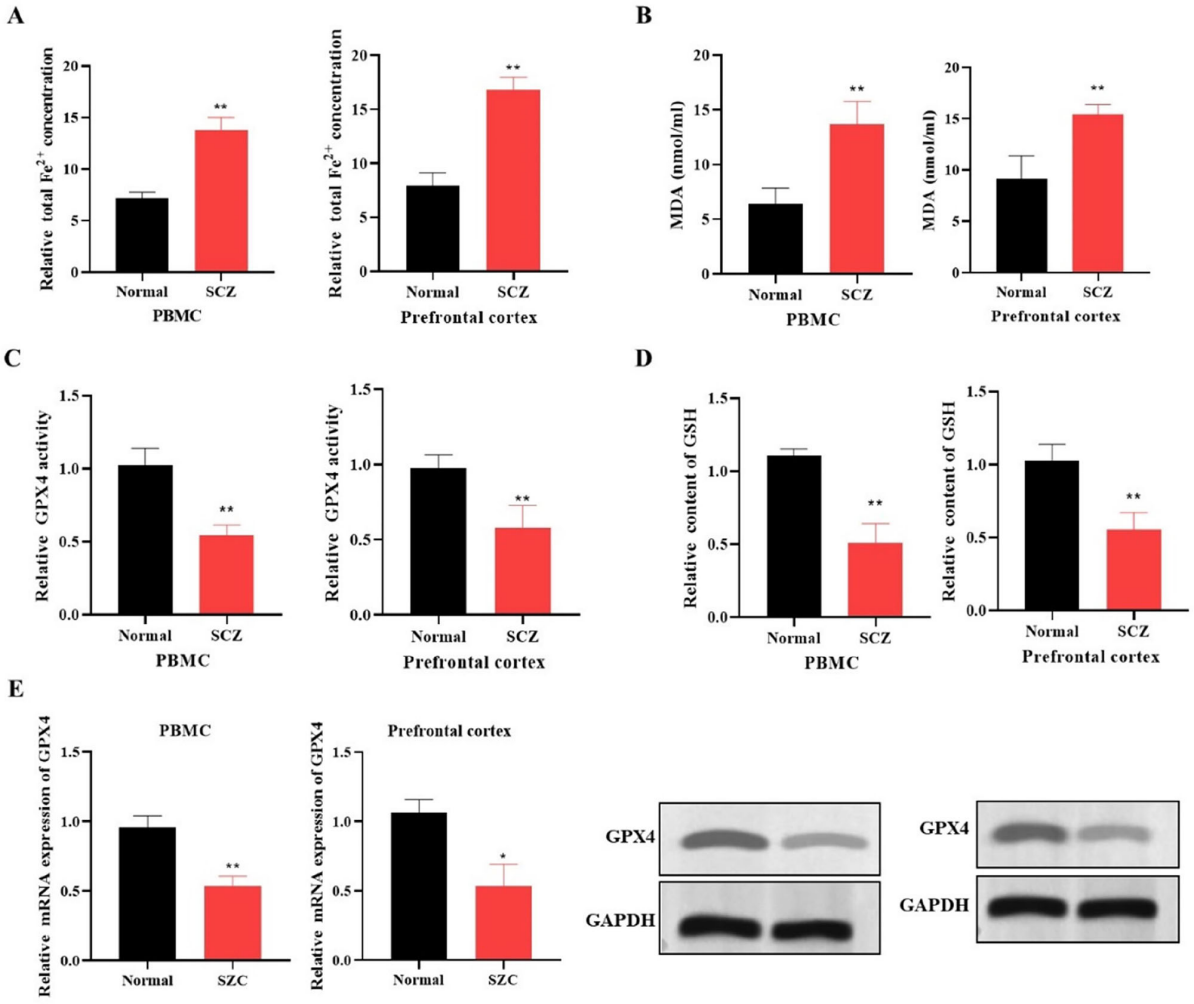
Ferroptosis is active in the prefrontal cortex and PBMCs of SCZ patients. (A) Intracellular Fe^2^⁺ content was measured in prefrontal cortex samples and PBMCs from SCZ patients using an iron content determination assay (*n* = 3). (B) An MDA assay was utilized to quantify MDA levels, which are indicative of oxidative stress and lipid peroxidation (*n* = 3). (C) A GSH assay kit was used to measure the levels of GSH (*n* = 3). (D) GPX4 activity, critical for protection against oxidative damage, was assessed with a GPX4 ELISA kit (*n* = 3). (E) GPX4 expression was measured using qRT‐PCR and Western blot (*n* = 3). *p < 0.05 and **p < 0.01 show statistical significance when compared to the normal group.

The intracellular ferrous iron (Fe^2^⁺) concentration in the PBMCs and prefrontal cortex of SCZ patients was significantly elevated compared to healthy controls, as measured by an iron content determination method (Figure 2A). Lipid peroxidation and oxidative stress were assessed by measuring MDA levels using an MDA assay kit, which revealed a marked increase in the SCZ group (Figure 2B). The activity of antioxidant GPX4 was quantified using a GPX4 ELISA kit. The SCZ group exhibited significantly reduced GPX4 activity (Figure 2C). Similarly, GSH levels, essential for cellular defense against oxidative stress, were found to be lower in SCZ patients, as detected by a GSH assay kit (Figure 2D). Furthermore, both RT‐PCR and Western blot analysis demonstrated reduced GPX4 mRNA expression in the SCZ group compared to controls (Figure 2E). These findings suggest an increased ferroptosis activity in SCZ patients, potentially contributing to neuronal injury observed in this disorder.

### 3.3 Dysregulation of Ferroptosis Pathway and Neuronal Markers in cINs Derived From hiPSCs of SCZ Patients

**FIGURE 3:**
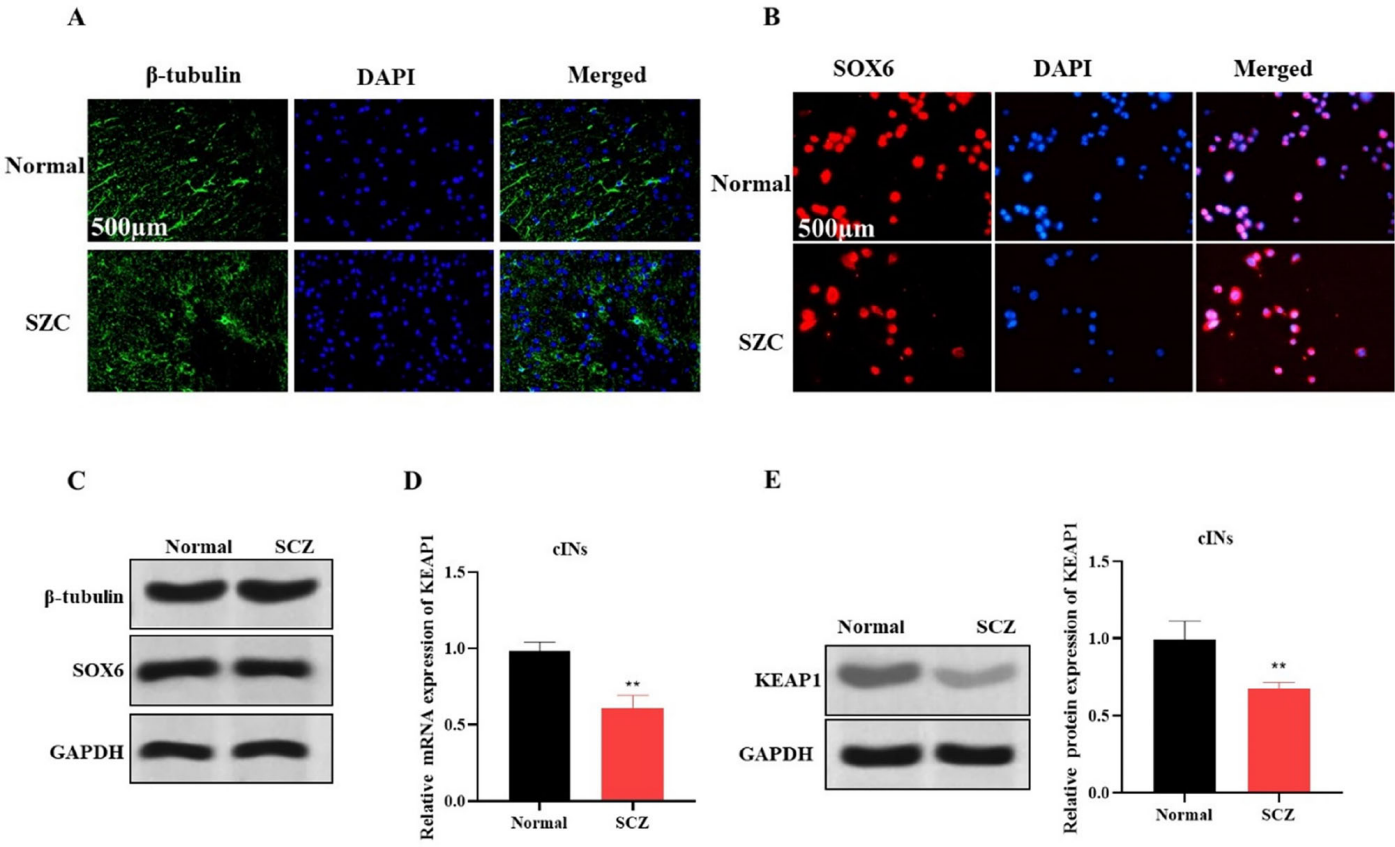
Dysregulation of ferroptosis pathway in cINs derived from hiPSCs of SCZ patients. (A–C) Immunofluorescence and Western blot showed expression of β‐tubulin and SOX6 in cINs derived from SCZ patients (*n* = 3). (D–E) qRT‐PCR and Western blot indicated a significant decrease in KEAP1 expression in SCZ‐derived cINs (*n* = 3). **p < 0.01 compared to normal group.

We next derived cINs from iPSCs of SCZ patient to explore alterations in the ferroptosis driver gene KEAP1 during neurodevelopment. To verify the neuronal nature, we first performed immunofluorescence and Western blot on cINs derived from SCZ patients for neuronal marker β‐tubulin and cINs SOX6 (Figure 3A–C). The results demonstrated the presence of these markers, thus, indicating successful isolation and development of cINs. qRT‐PCR and Western blots showed a reduction in KEAP1 expression in SCZ‐derived cINs (Figure 3D,E), indicating increased susceptibility to oxidative stress and ferroptosis. These findings highlight that reduced KEAP1 expression could still contribute to neuronal vulnerability and dysfunction in SCZ possibly through its involvement in the regulation of ferroptosis in developing neurons.

### 3.4 KEAP1 Regulates Ferroptosis in hiPSC‐Derived cINs

**FIGURE 4:**
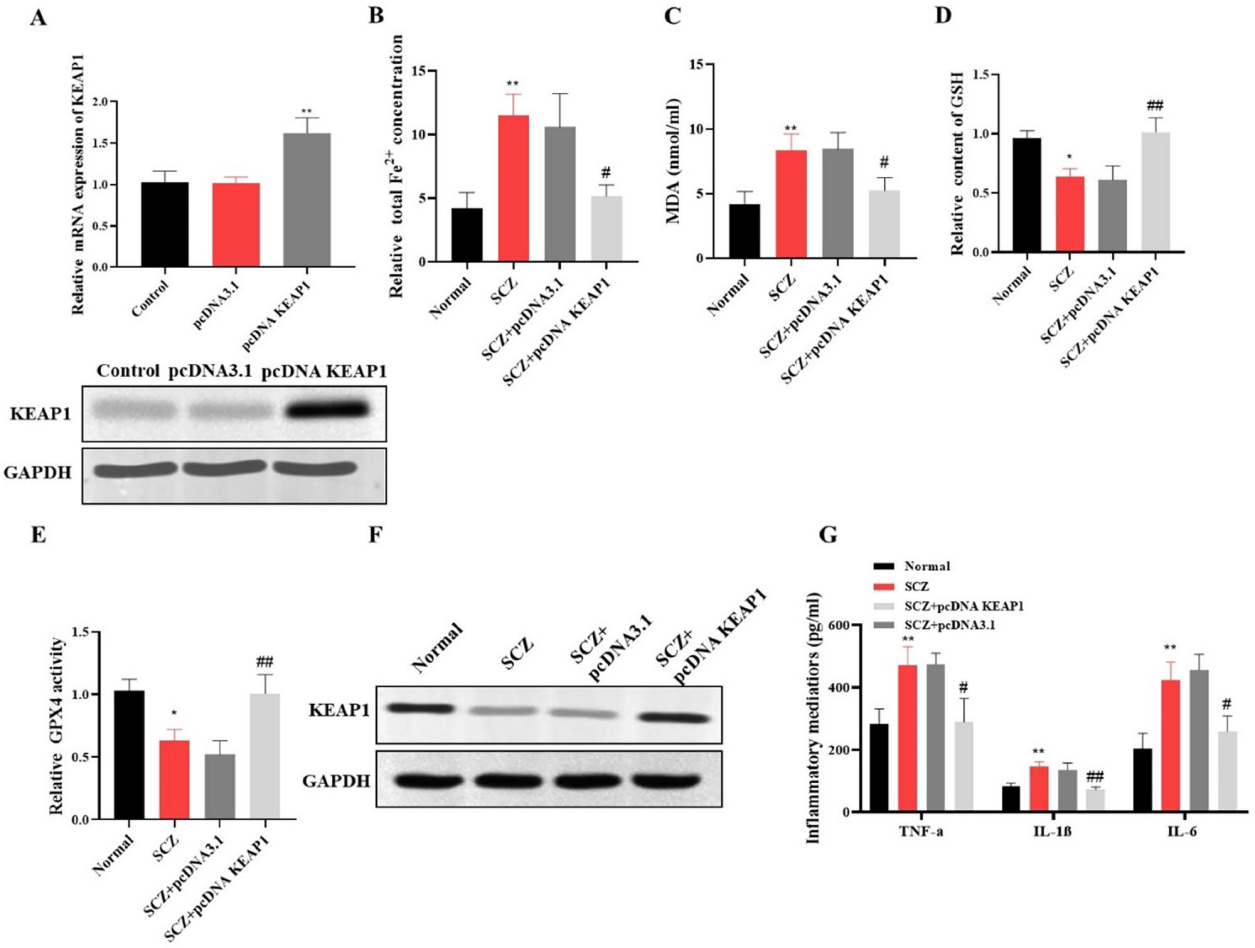
KEAP1 regulates ferroptosis in hiPSC‐derived cINs. (A) Validation of KEAP1 overexpression in cINs. (B) Quantification of Fe^2^⁺ levels showed a reduction in iron levels following KEAP1 overexpression. (C) Measurement of lipid peroxidation indicated a decrease in oxidative stress upon KEAP1 overexpression. (D) Assessment of GSH levels showed a restoration of antioxidant capacity in KEAP1‐overexpressing cells. (E) Measurement of GPX4 activity revealed increased GPX4 activity following KEAP1 overexpression. (F) Western blot analysis demonstrated increased KEAP1 protein expression in the KEAP1‐overexpressing group. (G) Quantification of inflammatory cytokines in cell culture supernatants showed a reduction in pro‐inflammatory mediators upon KEAP1 overexpression. *p < 0.05, **p < 0.01 versus the normal group; ^#^ *p* < 0.05, ^##^ *p* < 0.01 versus the SCZ group.

We next transfected cINs with KEAP1 to investigate their involvement in regulating ferroptosis and oxidative stress in hiPSC‐derived cINs. KEAP1 overexpression in cINs generated from SCZ patient hiPSCs was verified by Western blots and qRT‐PCR, both of which revealed KEAP1 overexpression following transfection with pcDNA KEAP1 (Figure 4A). In the SCZ group, elevated intracellular Fe^2^⁺ levels, a hallmark of ferroptosis, were significantly reduced following KEAP1 overexpression (Figure 4B). Similarly, the heightened levels of MDA observed in the SCZ group were lowered upon KEAP1 overexpression (Figure 4C). In contrast, the GSH levels, which were diminished in the SCZ group, showed a marked increase with KEAP1 overexpression, restoring cellular antioxidant capacity (Figure 4D). In addition, the activity of GPX4, which was reduced in the SCZ group, was significantly elevated after KEAP1 overexpression, further supporting the role of KEAP1 in protecting against ferroptosis (Figure 4E). KEAP1 expression, initially lower in the SCZ group, was restored to near‐normal levels with pcDNA KEAP1 transfection, as shown in both mRNA and protein analyses (Figure 4F). Finally, the elevated levels of TNF‐α, IL‐1β, and IL‐6, in the SCZ group were substantially reduced following KEAP1 overexpression, suggesting that KEAP1 may modulate neuroinflammation (Figure 4G). Overall, KEAP1 regulates ferroptosis and mitigates oxidative stress and inflammation in hiPSCs‐derived cINs.

### 3.5 KEAP1 Regulates Ferroptosis via the NRF2/HO‐1 Pathway in hiPSC‐Derived cINs

**FIGURE 5:**
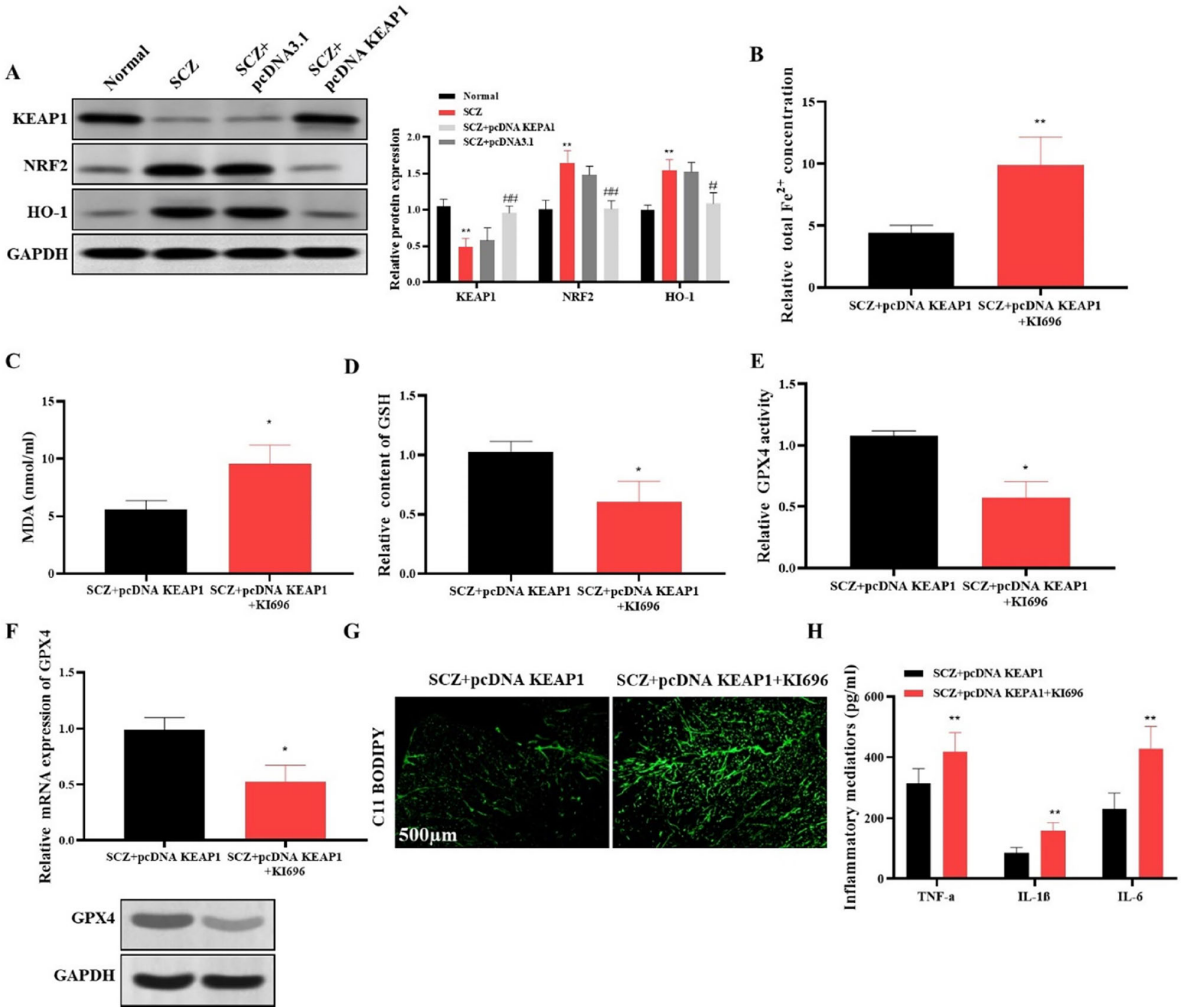
KEAP1 modulates ferroptosis through NRF2/HO‐1 pathway in hiPSC‐derived cINs. (A) Western blot showed the expression of NRF2 and HO‐1. (B) Quantification of Fe^2^⁺ levels, using an iron content determination assay. (C) Measurement of MDA levels using an MDA assay kit. (D) Quantification of GSH levels using a GSH assay kit. (E) Assessment of GPX4 activity using an ELISA kit. (F) GPX4 mRNA and protein were evaluated by qRT‐PCR and Western blot analysis, respectively. (G) Measurement of lipid oxidation using C11‐BODIPY staining, a dye specific for detecting lipid reactive oxygen species. (H) Quantification of inflammatory cytokines in the cell culture supernatant using ELISA kits. *p < 0.05 and **p < 0.01 show statistical significance when compared to SCZ‐pcDNA KEAP1 group.

KEAP1 regulates the activity of NRF2, a crucial transcription factor responsible for redox equilibrium. Given the importance of this signaling pathway in redox homeostasis (Li et al. 2021), we investigated its function in SCZ‐derived cortical cINs using KI696, a powerful probe that disrupts the interaction between KEAP1 and NRF2. Successful overexpression of KEAP1 was confirmed by Western blot, which also revealed increased expression of both NRF2 and HO‐1 in the SCZ group, indicating enhanced activation of this pathway (Figure 5A). In contrast, overexpression of KEAP1 significantly reduced the levels of NRF2 and HO‐1. To further assess the effects of disrupting the KEAP1–NRF2 interaction, we compared the SCZ+pcDNA KEAP1+KI696 group to the SCZ+pcDNA KEAP1 group. Disruption of KEAP1‐NRF2 signaling with KI696 led to marked alterations in several cellular parameters. Specifically, the SCZ+pcDNA KEAP1+KI696 group showed a significant increase in intracellular Fe^2^⁺ concentration (Figure 5B) and MDA levels (Figure 5C) and a reduction in GSH levels (Figure 5D), GPX4 expression (Figures 5E,F), and increased lipid oxidation as determined by C11‐BODIPY staining (Figure 5G). In addition, the inflammatory mediators were significantly elevated in the SCZ+pcDNA KEAP1+KI696 group compared to the SCZ+pcDNA KEAP1 group, suggesting that disruption of KEAP1‐NRF2 signaling exacerbates inflammation (Figure 5H). Overall, KEAP1‐NRF2/HO‐1 pathway regulates ferroptosis, oxidative stress, iron metabolism, and inflammation in SCZ‐derived cINs.

## 4 Discussion

SCZ is a complicated psychiatric condition that is impacted by genetic, neurodevelopmental, and environmental variables and affects around 1% of the global population (Hashimoto 2019). Emerging data suggest that oxidative stress and neuroinflammation are major contributions to the pathogenesis of SCZ (Ahmed et al. 2024; Upthegrove and Khandaker 2020). In this study, we identified an association between SCZ and reduced expression of KEAP1, a critical regulator of the NRF2/HO‐1 pathway, implicating its involvement in the disease's underlying mechanisms. SCZ is significantly influenced by oxidative stress, which is defined as an imbalance between reactive oxygen species production and antioxidant defenses (Afzal et al. 2023). Studies have consistently reported elevated oxidative stress levels in SCZ patients, marked by increased reactive oxygen species and decreased antioxidant capacity (Więdłocha et al. 2023). This study found increased intracellular Fe^2^⁺ concentration and MDA levels, both of which indicate heightened lipid peroxidation and oxidative stress in SCZ patients. This supports previous studies linking oxidative damage to the disorder (Jørgensen 2016).

Antioxidants such as GPX4 and GSH play essential roles in neutralizing oxidative damage. However, in our investigation, SCZ patients demonstrated decreased GPX4 activity and lower GSH levels, indicating an increased sensitivity to ferroptosis, a kind of cell death caused by iron‐dependent lipid peroxidation (Mohan et al. 2024; Y. Wang, Li, et al. 2024). Reduced antioxidant defenses, particularly in GPX4 activity, could contribute to neuronal injury in SCZ, as GPX4 is crucial for preventing ferroptosis in neurons (Ryan et al. 2023)

The KEAP1‐NRF2/HO‐1 pathway is a well‐known antioxidant defense mechanism (Huang et al. 2020; Kanwugu and Glukhareva 2023). Under normal conditions, KEAP1 sequesters NRF2 in the cytoplasm, promoting its degradation (Liu et al. 2023). However, oxidative stress disrupts this interaction, allowing NRF2 to translocate into the nucleus and activate antioxidant and cytoprotective genes such as HO‐1 (Sun et al. 2023). Dysregulation of this pathway is implicated in several neuropsychiatric disorders, including SCZ (Bhandari et al. 2021). cINs are inhibitory neurons and serve a key function in maintaining the balance between excitation and inhibition in the brain, with a particularly high concentration in the prefrontal cortex—an area closely associated to SCZ disease (Vid Prkačin et al. 2023). These interneurons, known for their rapid firing patterns and expression of the calcium‐binding protein parvalbumin, have become a key focus in SCZ research. Disruptions in parvalbumin‐expressing interneurons are thought to contribute to the cognitive deficits characteristic of SCZ by disturbing the brain's excitation–inhibition balance (Kaar et al. 2019; Toker et al. 2018). In this study, we used cINs derived from hiPSCs of SCZ patients to investigate the involvement of the KEAP1‐NRF2/HO‐1 pathway in the disorder. Our findings reveal that KEAP1 is downregulated in SCZ‐derived cINs, suggesting that disturbances in the KEAP1‐NRF2 pathway may serve as an early contributor to neuronal vulnerability in SCZ.

Furthermore, iron dysregulation is linked to ferroptosis, with iron excess promoting oxidative stress through Fenton reactions (Chung, Fish, and Lewis 2016). SCZ patients displayed elevated intracellular Fe^2^⁺ concentrations, supporting previous research suggesting that iron dysregulation contributes to the pathophysiology of the disorder (Lotan et al. 2023). The overexpression of KEAP1 reduced intracellular Fe^2^⁺ levels in hiPSC‐derived cINs, indicating its protective role against ferroptosis and iron‐mediated oxidative damage.

Inflammation is another critical factor in SCZ, and elevated levels of pro‐inflammatory cytokines have been widely reported in SCZ patients (Kim et al. 2023; Li and Zeng 2024). Our study revealed that the KEAP1–NRF2 interaction could be disrupted by KI696, leading to significant changes in pathways related to oxidative stress, iron metabolism, and inflammation. KI696 treatment exacerbated inflammatory responses suggesting that modulating KEAP1‐NRF2 signaling could influence both oxidative stress and inflammatory pathways in SCZ.

The main limitation of our study was that protective factors such as psychotherapy and counseling interventions, which may prevent or alleviate symptoms at onset, were not evaluated in this study. Future research should consider investigating the impact of such interventions in this context.

In conclusion, our study provides compelling evidence that KEAP1‐NRF2/HO‐1 pathway regulates ferroptosis, oxidative stress, and inflammation in SCZ. The dysregulation of KEAP1 in SCZ may contribute to neuronal injury, with ferroptosis acting as a potential mechanism. Targeting the KEAP1–NRF2 interaction, as demonstrated with KI696, offers a promising therapeutic approach, although further studies are needed to explore its efficacy in preclinical and clinical settings.

## Author Contributions

**Feng Zhu**: validation, methodology, software. **Zhifang Wang**: software, data curation, writing–original draft. **Shuguang Hua**: funding acquisition, project administration, supervision, resources.

## Ethics Statement

We submitted and received approval of this protocol (HU2022_GHXZ) from Hubei University of Science and Technology.

## Conflicts of Interest

The authors declare no conflicts of interest.

### Peer Review

The peer review history for this article is available at https://publons.com/publon/10.1002/brb3.70311.

## Data Availability

The data that support the findings of this study are available from the corresponding author upon reasonable request.

## References

[brb370311-bib-0001] Afzal, S. , A. S. Abdul Manap , A. Attiq , I. Albokhadaim , M. Kandeel , and S. M. Alhojaily . 2023. “From Imbalance to Impairment: The Central Role of Reactive Oxygen Species in Oxidative Stress‐Induced Disorders and Therapeutic Exploration.” Frontiers in Pharmacology 14: 1269581. 37927596 10.3389/fphar.2023.1269581 PMC10622810

[brb370311-bib-0002] Ahmed, G. K. , H. K.‐A. Ramadan , K. Elbeh , and N. A. Haridy . 2024. “The Role of Infections and Inflammation in Schizophrenia: Review of the Evidence.” Middle East Current Psychiatry 31: 9.

[brb370311-bib-0003] An, R. , D. Li , Y. Dong , et al. 2021. “Methylcobalamin Protects Melanocytes From H_2_O_2_‐Induced Oxidative Stress by Activating the Nrf2/HO‐1 Pathway.” Drug Design, Development and Therapy 15: 4837–4848. 34876806 10.2147/DDDT.S336066 PMC8643160

[brb370311-bib-0004] Bellezza, I. , I. Giambanco , A. Minelli , and R. Donato . 2018. “Nrf2‐Keap1 Signaling in Oxidative and Reductive Stress.” Biochimica et Biophysica Acta (BBA)—Molecular Cell Research 1865: 721–733. 29499228 10.1016/j.bbamcr.2018.02.010

[brb370311-bib-0005] Bhandari, R. , S. Kaur , and A. Kuhad . 2021. “The Nrf2 Pathway in Psychiatric Disorders: Pathophysiological Role and Potential Targeting.” Expert Opinion on Therapeutic Targets 25, no. 2: 115–139. 33557652 10.1080/14728222.2021.1887141

[brb370311-bib-0006] Chaves, C. , S. M. Dursun , M. Tusconi , and J. E. C. Hallak . 2024. “Neuroinflammation and Schizophrenia—Is There a Link?” Frontiers in Psychiatry 15: 1356975. 38389990 10.3389/fpsyt.2024.1356975 PMC10881867

[brb370311-bib-0007] Chen, M. Y. , Q. Zhang , Y. F. Liu , et al. 2023. “Schizophrenia and Oxidative Stress From the Perspective of Bibliometric Analysis.” Frontiers in Psychiatry 14: 1145409. 36923522 10.3389/fpsyt.2023.1145409 PMC10008861

[brb370311-bib-0008] Chung, D. W. , K. N. Fish , and D. A. Lewis . 2016. “Pathological Basis for Deficient Excitatory Drive to Cortical Parvalbumin Interneurons in Schizophrenia.” American Journal of Psychiatry 173: 1131–1139. 27444795 10.1176/appi.ajp.2016.16010025 PMC5089927

[brb370311-bib-0009] Guo, H. , Y. Liu , X. Yu , N. Tian , Y. Liu , and D. Yu . 2024. “Identifying Key Antioxidative Stress Factors Regulating Nrf2 in the Genioglossus With Human Umbilical Cord Mesenchymal Stem‐Cell Therapy.” Scientific Reports 14: 5838. 38462642 10.1038/s41598-024-55103-8 PMC10925593

[brb370311-bib-0010] Guo, W. , D. Huang , and S. Li . 2023. “Lycopene Alleviates Oxidative Stress‐Induced Cell Injury in Human Vascular Endothelial Cells by Encouraging the SIRT1/Nrf2/HO‐1 Pathway.” Clinical and Experimental Hypertension 45: 2205051. 37120838 10.1080/10641963.2023.2205051

[brb370311-bib-0011] Hashimoto, K. 2019. “Recent Advances in the Early Intervention in Schizophrenia: Future Direction From Preclinical Findings.” Current Psychiatry Reports 21: 75. 31278495 10.1007/s11920-019-1063-7

[brb370311-bib-0012] Huang, C. Y. , J. S. Deng , W. C. Huang , W. P. Jiang , and G. J. Huang . 2020. “Attenuation of Lipopolysaccharide‐Induced Acute Lung Injury by Hispolon in Mice, Through Regulating the TLR4/PI3K/Akt/mTOR and Keap1/Nrf2/HO‐1 Pathways, and Suppressing Oxidative Stress‐Mediated ER Stress‐Induced Apoptosis and Autophagy.” Nutrients 12, no. 6: 1742. 32532087 10.3390/nu12061742 PMC7352175

[brb370311-bib-0013] Jørgensen, A. 2016. “Antioxidants as Adjunctive Therapy in Schizophrenia.” Ugeskrift for Laeger 178: V68238. 27189103

[brb370311-bib-0014] Kaar, S. J. , I. Angelescu , T. R. Marques , and O. D. Howes . 2019. “Pre‐Frontal Parvalbumin Interneurons in Schizophrenia: A Meta‐Analysis of Post‐Mortem Studies.” Journal of Neural Transmission 126: 1637–1651. 31529297 10.1007/s00702-019-02080-2 PMC6856257

[brb370311-bib-0015] Kanwugu, O. N. , and T. V. Glukhareva . 2023. “Activation of Nrf2 Pathway as a Protective Mechanism Against Oxidative Stress‐Induced Diseases: Potential of Astaxanthin.” Archives of Biochemistry and Biophysics 741: 109601. 37086962 10.1016/j.abb.2023.109601

[brb370311-bib-0016] Kim, H. , S.‐H. Baek , J.‐W. Kim , et al. 2023. “Inflammatory Markers of Symptomatic Remission at 6 Months in Patients With First‐Episode Schizophrenia.” Schizophrenia 9: 68. 37794014 10.1038/s41537-023-00398-1 PMC10550944

[brb370311-bib-0017] Li, J. , K. Lu , F. Sun , et al. 2021. “Panaxydol Attenuates Ferroptosis Against LPS‐Induced Acute Lung Injury in Mice by Keap1‐Nrf2/HO‐1 Pathway.” Journal of Translational Medicine 19: 96. 33653364 10.1186/s12967-021-02745-1 PMC7927246

[brb370311-bib-0018] Li, Y. T. , and X. Zeng . 2024. “Circulating Inflammatory Cytokines Influencing Schizophrenia: A Mendelian Randomization Study.” Frontiers in Psychiatry 15: 1417213. 38979494 10.3389/fpsyt.2024.1417213 PMC11228335

[brb370311-bib-0019] Liu, N. , W. Yu , M. Sun , et al. 2024. “Research Trends and Hotspots of Ferroptosis in Neurodegenerative Diseases From 2013 to 2023: A Bibliometrics Study.” Heliyon 10: e29418. 38638970 10.1016/j.heliyon.2024.e29418 PMC11024616

[brb370311-bib-0020] Liu, Y. , S. Wang , G. Jin , et al. 2023. “Network Pharmacology‐Based Study on the Mechanism of ShenKang Injection in Diabetic Kidney Disease Through Keap1/Nrf2/Ho‐1 Signaling Pathway.” Phytomedicine 118: 154915. 37392674 10.1016/j.phymed.2023.154915

[brb370311-bib-0021] Lotan, A. , S. Luza , C. M. Opazo , et al. 2023. “Perturbed Iron Biology in the Prefrontal Cortex of People With Schizophrenia.” Molecular Psychiatry 28: 2058–2070. 36750734 10.1038/s41380-023-01979-3 PMC10575779

[brb370311-bib-0022] Luo, L. , F. Huang , S. Zhong , R. Ding , J. Su , and X. Li . 2022. “Astaxanthin Attenuates Ferroptosis via Keap1‐Nrf2/HO‐1 Signaling Pathways in LPS‐Induced Acute Lung Injury.” Life Sciences 311: 121091. 36252699 10.1016/j.lfs.2022.121091

[brb370311-bib-0023] McCutcheon, R. A. , R. S. E. Keefe , and P. K. McGuire . 2023. “Cognitive Impairment in Schizophrenia: Aetiology, Pathophysiology, and Treatment.” Molecular Psychiatry 28: 1902–1918. 36690793 10.1038/s41380-023-01949-9 PMC10575791

[brb370311-bib-0024] Mohan, S. , H. A. Alhazmi , R. Hassani , et al. 2024. “Role of Ferroptosis Pathways in Neuroinflammation and Neurological Disorders: From Pathogenesis to Treatment.” Heliyon 10: e24786. 38314277 10.1016/j.heliyon.2024.e24786 PMC10837572

[brb370311-bib-0025] Mosquera, F. E. C. , M. C. Guevara‐Montoya , V. Serna‐Ramirez , and Y. Liscano . 2024. “Neuroinflammation and Schizophrenia: New Therapeutic Strategies Through Psychobiotics, Nanotechnology, and Artificial Intelligence (AI).” Journal of Personalized Medicine 14, no. 4: 391. 38673018 10.3390/jpm14040391 PMC11051547

[brb370311-bib-0026] Ryan, S. K. , C. L. Ugalde , A.‐S. Rolland , J. Skidmore , D. Devos , and T. R. Hammond . 2023. “Therapeutic Inhibition of Ferroptosis in Neurodegenerative Disease.” Trends in Pharmacological Sciences 44: 674–688. 37657967 10.1016/j.tips.2023.07.007

[brb370311-bib-0027] Sawa, A. , and T. W. Sedlak . 2016. “Oxidative Stress and Inflammation in Schizophrenia.” Schizophrenia Research 176: 1–2. 27395767 10.1016/j.schres.2016.06.014

[brb370311-bib-0028] Schmitt, A. , P. Falkai , and S. Papiol . 2023. “Neurodevelopmental Disturbances in Schizophrenia: Evidence From Genetic and Environmental Factors.” Journal of Neural Transmission 130: 195–205. 36370183 10.1007/s00702-022-02567-5 PMC9660136

[brb370311-bib-0029] Sun, Y. Y. , H. J. Zhu , R. Y. Zhao , et al. 2023. “Remote Ischemic Conditioning Attenuates Oxidative Stress and Inflammation via the Nrf2/HO‐1 Pathway in MCAO Mice.” Redox Biology 66: 102852. 37598463 10.1016/j.redox.2023.102852 PMC10462885

[brb370311-bib-0030] Suzuki, T. , J. Takahashi , and M. Yamamoto . 2023. “Molecular Basis of the KEAP1‐NRF2 Signaling Pathway.” Molecules and Cells 46: 133–141. 36994473 10.14348/molcells.2023.0028 PMC10070164

[brb370311-bib-0031] Tandon, R. , H. Nasrallah , S. Akbarian , et al. 2024. “The Schizophrenia Syndrome, Circa 2024: What We Know and How That Informs Its Nature.” Schizophrenia Research 264: 1–28. 38086109 10.1016/j.schres.2023.11.015

[brb370311-bib-0032] Toker, L. , B. O. Mancarci , S. Tripathy , and P. Pavlidis . 2018. “Transcriptomic Evidence for Alterations in Astrocytes and Parvalbumin Interneurons in Subjects with Bipolar Disorder and Schizophrenia.” Biological Psychiatry 84: 787–796. 30177255 10.1016/j.biopsych.2018.07.010 PMC6226343

[brb370311-bib-0033] Upthegrove, R. , and G. M. Khandaker . 2020. “Cytokines, Oxidative Stress and Cellular Markers of Inflammation in Schizophrenia.” Current Topics in Behavioral Neurosciences 44: 49–66. 31115797 10.1007/7854_2018_88

[brb370311-bib-0034] Vid Prkačin, M. , I. Banovac , Z. Petanjek , and A. Hladnik . 2023. “Cortical Interneurons in Schizophrenia—Cause or Effect?” Croatian Medical Journal 64: 110–122. 37131313 10.3325/cmj.2023.64.110 PMC10183954

[brb370311-bib-0035] Wang, C. , S. Chen , H. Guo , et al. 2022. “Forsythoside A Mitigates Alzheimer's‐Like Pathology by Inhibiting Ferroptosis‐Mediated Neuroinflammation via Nrf2/GPX4 Axis Activation.” International Journal of Biological Sciences 18: 2075–2090. 35342364 10.7150/ijbs.69714 PMC8935224

[brb370311-bib-0036] Wang, L. , W. Lou , Y. Zhang , Z. Chen , Y. Huang , and H. Jin . 2023. “HO‐1‐Mediated Autophagic Restoration Protects Lens Epithelial Cells Against Oxidative Stress and Cellular Senescence.” Investigative Ophthalmology & Visual Science 64: 6. 10.1167/iovs.64.15.6 PMC10702788 38051262

[brb370311-bib-0037] Wang, X. , K. Li , T. Song , S. Xing , W. Wang , and Y. Fang . 2024. “Advances in Ferroptosis in Head and Neck Cancer (Review).” Biomedical Reports 21: 151. 39247426 10.3892/br.2024.1839 PMC11375624

[brb370311-bib-0038] Wang, X. , J. Liu , Z. Dai , and Y. Sui . 2021. “Andrographolide Improves PCP‐Induced Schizophrenia‐Like Behaviors Through Blocking Interaction Between NRF2 and KEAP1.” Journal of Pharmacological Sciences 147: 9–17. 34294378 10.1016/j.jphs.2021.05.007

[brb370311-bib-0039] Wang, Y. , H. Li , Q. He , R. Zou , J. Cai , and L. Zhang . 2024. “Ferroptosis: Underlying Mechanisms and Involvement in Neurodegenerative Diseases.” Apoptosis 29: 3–21. 37848673 10.1007/s10495-023-01902-9

[brb370311-bib-0040] Wang, Y. , S. Wu , Q. Li , H. Sun , and H. Wang . 2023. “Pharmacological Inhibition of Ferroptosis as a Therapeutic Target for Neurodegenerative Diseases and Strokes.” Advanced Science 10, no. 24: e2300325. 37341302 10.1002/advs.202300325 PMC10460905

[brb370311-bib-0041] Wei, R. , Y. Zhao , J. Wang , et al. 2021. “Tagitinin C Induces Ferroptosis Through PERK‐Nrf2‐HO‐1 Signaling Pathway in Colorectal Cancer Cells.” International Journal of Biological Sciences 17: 2703–2717. 34345202 10.7150/ijbs.59404 PMC8326123

[brb370311-bib-0042] Weiss‐Sadan, T. , M. Ge , M. Hayashi , et al. 2023. “NRF2 activation Induces NADH‐Reductive Stress, Providing a Metabolic Vulnerability in Lung Cancer.” Cell Metabolism 35: 487–503. 36841242 10.1016/j.cmet.2023.01.012 PMC9998367

[brb370311-bib-0043] Więdłocha, M. , N. Zborowska , P. Marcinowicz , et al. 2023. “Oxidative Stress Biomarkers Among Schizophrenia Inpatients.” Brain Sciences 13, no. 3: 490. 36979300 10.3390/brainsci13030490 PMC10046541

[brb370311-bib-0044] Winship, I. R. , S. M. Dursun , G. B. Baker , et al. 2019. “An Overview of Animal Models Related to Schizophrenia.” Canadian Journal of Psychiatry [Revue Canadienne De Psychiatrie] 64: 5–17. 29742910 10.1177/0706743718773728 PMC6364139

[brb370311-bib-0045] Xu, Y. , B. Jia , J. Li , Q. Li , and C. Luo . 2024. “The Interplay Between Ferroptosis and Neuroinflammation in Central Neurological Disorders.” Antioxidants 13, no. 4: 395. 38671843 10.3390/antiox13040395 PMC11047682

[brb370311-bib-0046] Yi, M. , L. Cruz Cisneros , E. J. Cho , et al. 2024. “Nrf2 Pathway and Oxidative Stress as a Common Target for Treatment of Diabetes and Its Comorbidities.” International Journal of Molecular Sciences 25, no. 2: 821. 38255895 10.3390/ijms25020821 PMC10815857

[brb370311-bib-0047] Zhang, J. C. , W. Yao , C. Dong , M. Han , Y. Shirayama , and K. Hashimoto . 2018. “Keap1‐Nrf2 Signaling Pathway Confers Resilience Versus Susceptibility to Inescapable Electric Stress.” European Archives of Psychiatry and Clinical Neuroscience 268: 865–870. 29119264 10.1007/s00406-017-0848-0

